# Holistic Health Status Questionnaire: developing a measure from a Hong Kong Chinese population

**DOI:** 10.1186/s12955-016-0416-8

**Published:** 2016-02-25

**Authors:** Choi Wan Chan, Frances Kam Yuet Wong, Siu Ming Yeung, Fok Sum

**Affiliations:** School of Nursing, The Hong Kong Polytechnic University, Hung Hom Kowloon, Hong Kong; Nethersole Institute of Continuing Holistic Health Education, Room J-7-28, 11 Chuen On Road, Tai Po, NT Hong Kong

**Keywords:** Holistic Health Status Questionnaire, Holism, Holistic health, Instrument development, Factor analysis, Hong Kong Chinese

## Abstract

**Background:**

The increased prevalence of chronic diseases is a global health issue. Once chronic disease is diagnosed, individuals face lifelong healthcare treatments, and the disabilities and disturbances resulting from their illness will affect the whole person. A valid tool that can measure clients’ holistic care needs is important to enable us to identify issues of concern and address them early to prevent further complications. This study aimed to develop and evaluate the psychometric properties of a scale measuring holistic health among chronically ill individuals.

**Methods:**

The research was an instrument development and validation study using three samples of Hong Kong Chinese people. The first sample (*n* = 15) consisted of stroke survivors who had experienced disruption of their total being, and was used as a basis for the generation of scale items. In the second and third samples (*n* = 319, *n* = 303), respondents with various chronic illnesses were assessed in order to estimate the psychometric properties of the scale. A total of 52 items were initially generated, and 7 items with a factor loading less than 0.3 were removed in the process, as substantiated by the literature and expert panel reviews.

**Results:**

Exploratory factor analysis identified a 45-item, 8-factor Holistic Health Status Questionnaire (HHSQ) that could account for 56.38 % of the variance. The HHSQ demonstrated content validity, acceptable internal consistency (0.59–0.92) and satisfactory convergent validity from moderate to high correlation with similar constructs (*r* ≥ 0.46, *p* < 0.01).

**Conclusions:**

The HHSQ tapped into the relational experiences and connectedness among the bio-psycho-social-spiritual dimensions of a Chinese person with chronic disease, with acceptable psychometric properties.

## Background

Chronic diseases account for approximately 60 % of population mortality globally [1]. The World Health Organization projects that of 64 million people who will die in 2015, 41 million will die of a chronic disease [2]. In Hong Kong (HK), approximately 61 % of total registered deaths in 2006 were attributed to chronic diseases, including heart diseases, stroke, respiratory diseases and cancer [2]. Over 70 % of community-dwelling elderly people in HK suffer from at least one kind of chronic disease [3]. Chronic diseases of the circulatory system, respiratory system and neoplasm accounted for at least 32 % of the total allocated health expenditure in Hong Kong during the 2012 financial year [2]. At the individual level, in the case of a chronic disease like stroke, almost 40 % of survivors are left with at least moderate disability [4–6] and become highly dependent on caregivers for help in daily activities on a long-term basis [7, 8].

People who are chronically ill can experience an array of sudden loss of bodily functions [9–11]; psychological and emotional distress [12–14]; the loss of role and self-identity [12] leading to significant disruption in individual, family and social life [15]; and concerns for the future, sustaining hope, ability to heal, and maintaining spiritual strength [16]. Individuals can be viewed as being in multi-faceted relationships, and illness can be considered as a disturbance to these relationships. These relationships involve the connections between body, mind and transcendence, the nexus of the ecological, physical, familial, social and political environments [17]. Hence, these relational disturbances/experiences typically allude to the fact that the bio-physical, psychological, social and spiritual dimensions are not separate from the experiences of an individual as a whole, when that individual is chronically ill [18, 19]. Once chronic disease is diagnosed, individuals face lifelong healthcare treatments, and their disabilities and disturbances resulting from the illness fundamentally require long-term whole-person healthcare support. Among the chronically ill groups, stroke survivors tend to have more identified negative effects in terms of physical impairment [20], as well as psychological and social impacts [15, 21, 22]. A Danish study reported that 23.6 % of stroke patients purchased antidepressants during follow-up, a much higher rate than in the comparison osteoarthritis group, which documented a rate of 9.1 % [23]. Holistic healthcare attends to all the disturbed relationships of an ill person as a whole, even though the person is not thereby completely resilient to perfect wholeness. A holistic framework enabling healthcare to address all aspects of bio-physical, psychological, social and spiritual support in the management of chronic diseases is obviously needed.

The fundamental dimensions of holism encompass physical, psychological, social and spiritual aspects [24]. Holism in nursing “involves identifying the interrelationships of the bio-psycho-social-spiritual dimensions of a person” [25]. These aspects of holism essentially provide a generic framework for addressing the holistic well-being and concerns experienced by individuals with chronic disease during their long-term care.

To enhance holistic healthcare for chronic diseases in a Chinese population, the cultural attributes that underpin the generic holistic framework appear to be underexplored. Culturally, the concept of holism in Chinese societies is derived from the integrated beliefs of Confucianism, Buddhism and Taoism [26–29], which assert the importance of maintaining a balanced and holistic mind-body state. The other attribute of holism in Chinese culture is that the individual self is highly related to the family context. Close interpersonal relationships such as the emotional bond with family members are salient in Chinese culture [30]. Family connectedness becomes especially important when the family assumes an important role in taking care of a member who is experiencing illness. During the course of illness, Chinese traditionally believe that health and the associated interpersonal outcomes are related to fate, transcendent forces or the predestiny of the virtuous and evil-doers, with strong psychological feelings of guilt and self-blame [29, 31].

As holistic healthcare for chronic disease requires attention to the bio-psycho-socio-spiritual disturbances and concerns of individuals, addressing and assessing holistic well-being and concerns experienced by the individual is where the task begins. Research has been conducted to develop and test holistic health measures [32–34], but very little has focused on the Chinese population with chronic illnesses. Researchers embarking on the development of holistic health measures share some common world views. Existing well-established health status instruments such as the SF Quality of Life measures view health as compartmentalized into physical, mental, social and spiritual dimensions, rather than as a holistic entity [35]. Holistic health researchers assume that respondents perceive all aspects of the self as interrelated, thus expressing the self as a gestalt [36]. The expressions are subjective, so the use of the language in the items should be grounded in the experience of these individuals and reflect their responses. The generation of items for holistic measures therefore often begins with qualitative interviews to capture the stakeholders’ perspectives and descriptions in the experience of their health state [35, 37]. During this process, clinicians provide comments on the components, which help validate the statements [36]. The instrument is then subjected to reliability and validity testing.

There is a dearth of valid instrumentation addressing holistic health and well-being, particularly among Chinese people with chronic diseases. For health status measurements designed and tested in Western culture(s), difficulties were encountered in producing corresponding cultural and linguistic expressions in Chinese equivalent to the item wordings [38, 39]. Similarly, differences in health status measurement results between Western and Eastern cultures are widely recognized [38–42]. Embedded differences in cross-cultural interpretations include health status measurement domains, particularly those related to social functioning [38, 42], mental health [39, 42], physical functioning [39], and vitality, for measuring both physical and mental energy as well as fatigue [38, 39, 42]. In addition, for generic health status measurements, others have suggested that the domain of family functioning might be needed to acknowledge the impact of health on family life in cultures in which family life might play a more central role in individuals’ lives [39, 42]; the domain for spiritual aspects also appears lacking [43]. In view of the gaps in instrumentation, the purpose of this study was to develop an instrument with culturally-sensitive attributes that tap into Chinese people’s holistic health and well-being concerns in relation to chronic diseases. The conceptual framework of bio-psycho-social-spiritual holism was used to guide the present study.

## Methods

This research was a methodological study used to assess the validity (content/construct validity) and reliability of an instrument [44]. The study consisted of two stages. The first pertained to scale development, including the generation of scale items and the establishment of the content validity of the scale. The second phase aimed to establish the psychometric properties of the Holistic Health Status Questionnaire (HHSQ) that was developed in the first stage. The psychometric analysis involved construct validity and reliability in terms of evaluating the internal consistency of the scale using Cronbach’s α coefficients. The construct validity included a factor analysis and convergent validity. In convergent validity, it was anticipated that the constructs identified in HHSQ would be correlated with the SF-12 Health Survey (a quality of life measurement) based on the premise that an individual with a higher level of holistic health status is expected to enjoy a better quality of life.

### Stage 1: development of the HHSQ

The items of the HHSQ were first generated among post-stroke patients involved in a randomized controlled trial that included the measurement of holistic well-being as one of the outcomes. Stroke survivors were selected as the target group for item generation because they tend to experience all of the potential impacts of a chronic illness, including physical [20], psychological [23], social [21] and spiritual [9] dimensions. Semi-structured, face-to-face individual interviews with 15 discharged post-stroke patients were conducted to explore the holistic concerns of these patients within the first week of returning home after an acute episode involving a physical consult. The participants were selected by a purposive sampling method based on the following inclusion criteria: (1) Chinese, (2) aged >18 years, (3) first stroke, (4) cognitive status assessed by the Mini-Mental Status Examination with scores >21 [45], (5) able to communicate, (6) no debilitating co-morbidity, and (7) discharged home. Patients diagnosed with Transient Ischemic Attacks or with co-existing mental disorders were excluded from the study. This sample consisted of participants recruited from the stroke unit of a general hospital in Hong Kong, with ages ranging from 53 to 79 (mean (M) = 64.7, standard deviation (SD) = 8.9); seven were male and eight were female. All had received education at primary level or below, and 33.3 % described themselves as having a religious background, such as Buddhism (26.7 %) or Christianity (6.6 %). Almost all were supported by family caregivers. The average length of hospitalization was 13 days. All informants experienced residual problems, such as limb weakness, memory loss, dizziness, fatigue and sleep disturbances. During the interview, the start-up interview question “Would you please describe the event(s) directly connected with your stroke experience?” was asked, and follow-up questions were then posed to enable further exploration of participants’ initial answers [46]. The interviews were audio-taped and transcribed by a research assistant, and the accuracy of the transcripts was checked by a research team investigator. Data saturation was achieved when no information could be identified that added to new content units or themes [46]. The research team investigator analyzed all the qualitative data using content analysis. Units of content carrying the same meaning were crosschecked, discussed and eventually collapsed into themes, with consensus achieved with the other three research team investigators in multiple meetings. A total of 51 items representing the five aspects of concern in the physical, psychological, social, spiritual and cultural domains emerged from the data. These items were then reviewed by a panel of 5 experts including 1 nurse manager (medical), 1 specialty nurse (stroke), 1 community nurse, 1 holistic care educator and 1 researcher in the field of post-discharge and/or stroke care. The content validity index (CVI) was used to rate the content relevance of the items on a four-point scale: 1 = completely irrelevant, 2 = not relevant, 3 = relevant, and 4 = completely relevant. The experts reviewed the items independently. The CVI was calculated using the percentage of total items rated by the experts as either 3 or 4, a CVI rating above 0.8 being considered valid [47]. Besides the rating, the experts suggested rewording the sentences in 8 of the items to make the sentences more comprehensible. Apart from the 51 items, one of the panel experts suggested adding a new item, ‘I feel pain all over my body’, which was observed to be a common complaint in clinical encounters. The research team revisited the data and found that this complaint of pain all over the body had been mentioned by a number of informants but subsumed under the overall discomfort item. After discussion and considering the clinical reality, this item of ‘pain all over the body’ was included as one of the items. The revised version of the questionnaire was again sent to the expert panel for review. All scale items were judged as content valid with a score of either 3 or 4 in the second round of the expert review.

### Stage 2: psychometric properties of the HHSQ

The HHSQ developed in Stage 1 generated items that contained direct expressions of holistic concern after an episode of illness. Interestingly, these items conveyed no specific description of the illness concerned. The research team then asked if the HHSQ would be applicable to a population with residual health concerns other than stroke. We therefore selected two samples with known chronic illnesses for further testing.

#### Participants

Two independent samples of participants with chronic illnesses were recruited to establish the psychometric properties of the HHSQ. Sample 1 reported chronic illnesses, but no specific illness type was used to identify the factor structure and establish the scale reliability. Subjects were adults recruited from general health or social service settings. In sample 2, we deliberately chose patients from 3 chronic disease groups who were regularly followed up in the daycare service centers or hospital outpatient departments. These groups of chronically ill patients were used to evaluate the relationship between SF12 and HHSQ in testing the convergent validity of the instrument. The research assistant read the items to the respondents and helped them fill in the questionnaire, so the literacy of the respondents was not a concern in data collection. The instrument took 30 min to complete. A minimum of 260 subjects was considered adequate based on the recommended requirement of at least 5 participants per item for the psychometric assessment of an instrument [48].

##### Sample 1: identify factor structure and estimate scale reliability

A convenience sample of 319 adult Chinese participants recruited from a regional cluster of healthcare settings, including a regional hospital (22.0 %), a day rehabilitation center (6.0 %), a nursing home (20.3 %), and an elderly center (51.7 %), was used to identify the scale factor structure and establish the internal consistency reliability. A total of 345 subjects were approached, resulting in a response rate of 92.6 %. The sample age ranged from 21 to 99 years (M = 76.7, SD = 10.4), and 67.7 % were female. All participants had reported chronic illnesses, with 60.5 % having ≥ 2 types of chronic disease, such as stroke, coronary disease, diabetes, hypertension, renal failure or chronic obstructive pulmonary disease (COPD). The majority (72.3 %) had primary level education, and 71.8 % reported having a religious background.

##### Sample 2: perform convergent validity

A convenience sample of 303 adult Chinese participants with specific chronic diseases including COPD (*n* = 91, 30.0 %), diabetes (*n* = 110, 36.3 %), and chronic renal failure (*n* = 102, 33.7 %) was recruited from a medical unit of a regional hospital (5.0 %), daycare service centers (58.7 %) and a hospital outpatient department (36.3 %). Three hundred and eight subjects were approached, resulting in a response rate of 98.4 %. The sample age ranged from 18 to 91 years (M = 61.0, SD = 13.1), and 37.6 % were female. More than half of the sample (56.8 %) had secondary level education, and the majority (71.8 %) reported having a religious background.

### Procedures

Ethical approval to conduct the study was obtained from the Hong Kong Polytechnic University Human Subjects Ethics Subcommittee and Joint Chinese University of Hong Kong-New Territories East Cluster Clinical Research Ethics Committee. The study’s nature and purpose were explained to the participants by a research team member. They were assured of their privacy and anonymity. Each participant who agreed to take part in the study was asked to sign an informed consent, complete the HHSQ, HK-specific SF-12 and provide demographic information and the history of their chronic disease.

### Measures

#### The HHSQ

After expert review for content validity, the initial HHSQ consisted of 52 items. Each item was rated on a 4-point scale ranging from “none of the time” to “all of the time”. The HHSQ scores were obtained by summing the item scores. A higher score indicated better holistic well-being.

#### The SF-12 Health Survey

The SF-12 Health Survey [49] was developed as a shorter version of the SF-36 Health Survey. The SF-12 consists of 12 items in two domains: the physical and mental component summaries (PCS and MCS). The 12 items include two from each of the Physical Functioning, Role-Physical, Role-Emotional and Mental Health subscales, and one from each of the Bodily Pain, General Health, Vitality and Social Functioning subscales of the SF-36. The original SF-12 Health Survey has been translated, validated and shown to be reliable for use in a HK Chinese population [50, 51]. A higher score indicates better quality of life on the SF-12.

### Statistical analyses

Data were analyzed using the IBM SPSS Statistics software, version 20. Descriptive statistics were used to summarize the demographic data. An exploratory factor analysis (EFA) using principal component analysis (PCA) and varimax rotation was performed to examine the factor structure of the HHSQ. A Kaiser-Meyer-Olkin (KMO) score >0.6, which indicates adequate sample size for the factor analysis, and a significant value for Bartlett’s test of sphericity were employed to determine the factorability of the data [52]. The scree plot analysis and Kaiser-Guttman criterion with eigenvalues greater than 1.0 as the criteria were used to extract factors. Factor loadings that exceeded the criterion of 0.30 were used as a cut-off point to retain the significant items [53]. The internal consistency of the HHSQ was assessed through Cronbach’s α coefficients, with a value greater than 0.60 considered acceptable for a newly-developed instrument [54]. For convergent validity, Pearson’s correlation was used. A 0.05 level of significance was employed.

## Results

### Exploratory factor analysis

The significant value of Bartlett’s test of sphericity (*p* <.001) and the KMO value of 0.89 indicated that the assumptions for factor analysis were met. Initial exploratory factor analysis using eigenvalues greater than 1.0 yielded 12 factors, which accounted for 61.11 % of the total explained variance. Based on the scree plot, a discontinuity of the steep slope was around 7 to 9 factors, where the ‘elbow’ of the scree plot was located in this leveled-off area. From factor 7 to factor 12, the eigenvalues were 1.47, 1.30, 1.16, 1.13, 1.08, 1.06, respectively. It was decided to extract an eight-factor structure model, as this provided the best interpretation of items in terms of congruency and relevancy, as well as contributing a reasonable total variance (8-factor 52.59 % versus 7-factor 50.09 % and 9-factor 54.83 %). When examining the 8-factor model, item deletion was executed based on a number of considerations. These items were below the factor loading cut-off criterion of 0.3, with communalities <0.20, and with multiple loadings. We also examined the item-domain correlations to determine whether the Cronbach’s alpha changed with the removal of the item. The research team then considered the conceptual congruence of the item with the factors. Finally, seven items were removed from the initial 52-item HHSQ. One item, ‘I don’t get good sleep’, was retained in spite of a low factor loading (0.23) under the factor of physical symptoms, as sleeplessness had emerged from the qualitative data as a common and frequent concern and was conceptually relevant to the factor characteristic. The factor analysis was re-run after item deletion.

The final 8-factor, 45-item HHSQ accounted for 56.38 % of total variance (Table 1). The 8 factors were (1) psychological expression (12 items, item factor loadings ranging from 0.54 to 0.72), (2) changes in self and family (seven items, 0.40–0.74), (3) physical symptoms (ten items, 0.27–0.63), (4) social and family connectedness (three items, 0.51–0.85), (5) fatalism (three items, 0.78–0.88), (6) religion and faith (three items, 0.32–0.90), (7) self-query (four items, 0.45–0.63), and (8) coping style (three items, 0.63–0.83). The final eigenvalues (and variances) of the factors were 11.46 (25.48 %), 3.37 (7.50 %), 2.29 (5.09 %), 2.16 (4.81 %), 2.04 (4.52 %), 1.44 (3.21 %), 1.40 (3.10 %), and 1.21 (2.68 %), respectively. Based on the conceptual relevancy of the factor, the item ‘I don’t get good sleep’ in the final model achieved a factor loading of 0.27, close to the set criterion, and thus remained in factor 3-physical symptoms, as initially decided. There were 18 complex items carrying a factor loading that would allow them to be placed in more than one factor. Thirteen of these items were placed in the factors where they had the highest loadings. The remaining five items were housed in the host factors where they fit conceptually and bore a reasonable loading. These items included ‘I feel that my illness is causing trouble to my family’ (factor loading 0.40) under the factor of changes in self and family; ‘There is hope in my future’ (0.32) under religion and faith; ‘I ask, “Why do I have this illness?”’ (0.45) under self-query; and ‘There is something wrong with my body’ (0.36) and ‘I feel weak in my body/limbs’ (0.42) under physical symptoms.

**Table 1:** Rotated component matrix of the 45-item, 8-factor Holistic Health Status Questionnaire

Item	Factor							
	1	2	3	4	5	6	7	8
My mood is delighted.	**.723**	.174	.040	.405	.038	.074	.030	.000
I feel happy.	**.683**	.178	−.004	.346	−.045	.144	.017	.054
I feel very frightened.	**.682**	.045	.200	−.107	.074	−.058	.009	.023
I feel confused.	**.669**	.157	.313	.116	.134	−.072	.208	.021
With this illness, I cannot take care of myself.	**.664**	.196	.079	.001	.058	−.071	−.086	.009
My heart feels heavy.	**.659**	.134	.251	.198	.119	−.043	.096	.023
I feel sad.	**.657**	.151	.260	.206	.121	−.098	.253	−.019
I am really afraid that my illness will get worse.	**.617**	.138	.227	−.017	.057	−.071	.155	.032
I feel really uncomfortable emotionally.	**.595**	.213	.190	.216	.129	−.052	.263	.067
I have peace in my heart.	**.592**	.107	.083	.430	.026	.037	.041	.138
I feel that my illness is causing trouble to my family.	.566	**.397**	.037	−.221	.183	−.094	.026	.016
Living with this illness is hard.	**.563**	.339	.222	.132	.201	−.066	.031	.064
I want to cry.	**.535**	.059	.262	.140	.084	−.180	.124	.076
There is something wrong with my body.	.517	.053	**.363**	−.056	.008	.088	.151	−.130
I ask, “Why do I have this illness?”	.507	.097	.106	−.079	.109	−.078	**.446**	−.015
I feel weak in my body / limbs.	.434	.220	**.415**	.017	−.042	−.093	.026	−.023
It is not the same any more at work.	.134	**.739**	.184	−.019	.076	−.101	.058	−.010
I feel different than before when at home.	.180	**.669**	.153	.159	.073	−.109	.083	−.012
With this illness, I feel I have lost all my freedom.	.345	. **637**	−.007	.112	.081	.034	.176	−.021
I have become more clumsy than before.	.166	**.612**	.335	−.031	.165	.033	−.141	−.129
I do not go out as often as before.	.309	**.593**	−.002	−.027	−.140	.039	.212	−.057
It is a burden for my family to take care of me.	.416	**.467**	−.147	−.369	.081	.043	.079	.104
I feel dizzy.	.285	.037	**.628**	.044	−.010	−.043	−.055	.161
I feel pain all over my body.	.264	−.013	**.596**	.132	−.073	.001	.060	−.027
My appetite has worsened.	.021	.384	**.533**	.014	.057	−.036	.239	.061
I feel uncomfortable all over my body.	.483	.161	**.531**	.023	−.089	−.042	.108	−.041
I feel really tired.	.315	.244	**.526**	.043	.055	−.184	−.031	−.112
My memory has worsened.	.142	.130	**.516**	.144	.177	.014	−.254	−.109
I get headaches.	.385	−.086	**.480**	.119	−.031	−.056	.189	.125
My family gives me great comfort.	.131	−.102	.195	**.848**	−.013	.050	.025	.009
My family cares a lot about me.	.131	−.085	.141	**.834**	.004	.044	.010	.093
My friends give me great comfort.	.165	.192	−.060	**.511**	−.074	.037	−.048	.002
There is hope in my future!	−.007	.102	.312	.346	.231	**.323**	−.180	−.057
I believe that it is predestined!	.151	.093	.048	−.026	**.880**	−.050	.138	−.096
I believe it is fate.	.171	.068	.011	−.024	**.861**	−.039	.117	−.044
I think this is divine intervention.	.113	.058	−.035	−.028	**.775**	−.044	.173	−.055
I believe the Heavens/God/my religion is taking care of me.	−.109	−.052	−.071	.091	−.075	**.895**	−.096	.037
I find it helpful to pray to God/gods/my religion.	−.096	−.084	−.116	.048	−.090	**.884**	−.031	−.013
I’ve caused my own illness!	.221	.144	−.058	−.064	.233	.078	**.625**	.041
I still have much unfinished business.	.333	.184	.014	−.064	.038	−.074	**.549**	.112
I believe that this is karma!	.008	.015	.064	.068	.296	−.143	**.517**	−.011
I don’t get good sleep.	.100	.151	**.272**	.238	−.028	−.155	.287	−.236
So I am sick, anyway I have to accept it .	−.032	.008	.066	.053	.041	.071	.085	**.830**
I can’t help getting sick, so I should not think too much about it.	.108	−.089	−.054	−.048	−.049	−.181	−.134	**.685**
I think, “Let it be!”	.087	.002	.000	.091	−.186	.098	.130	**.634**
Eigenvalues	11.46	3.37	2.29	2.16	2.04	1.44	1.40	1.21
% of variance by factor	25.48	7.50	5.09	4.81	4.52	3.21	3.10	2.68

Items of respective factors with factor loadings bold and underlined. 1 psychological expression, 2 changes in self and family, 3 physical symptoms, 4 social and family connectedness, 5 fatalism, 6 religion and faith, 7 self-query, 8 coping style

### Reliability

The subscales’ α from factor 1 to factor 8 were 0.92, 0.82, 0.81, 0.73, 0.88, 0.64, 0.62, and 0.59 respectively. The item-to-subscale correlations were 0.58–0.75 (factor 1), 0.49–0.62 (factor 2), 0.31–0.64 (factor 3), 0.30–0.73 (factor 4), 0.67–0.84 (factor 5), 0.17–0.66 (factor 6), 0.27–0.47 (factor 7), and 0.34–0.50 (factor 8) (Table 2).

**Table 2:** Reliability statistics

Factor	Cronbach’s α	Item-to-subscale correlation	Subscale-to-total correlation
1 Psychological expression	0.92	0.58–0.75	0.92
2 Changes in self and family	0.82	0.49–0.62	0.73
3 Physical symptoms	0.81	0.31–0.64	0.80
4 Social and family connectedness	0.73	0.30–0.73	0.39
5 Fatalism	0.88	0.67–0.84	0.39
6 Religion and faith	0.64	0.17–0.66	0.02
7 Self-query	0.62	0.27–0.47	0.59
8 Coping style	0.59	0.34–0.50	0.12

### Convergent validity

Significant and moderate correlations were found between the total HHSQ scores and the scores of the two domains of the SF-12: the Physical Component Summary, PCS (0.46, *p* < 0.01) and the Mental Component Summary, MCS (0.70, *p* < 0.01). Significant but weak correlations (0.2) were also found between all of the subscale scores of the HHSQ and the domains of SF-12 (Table 3), except between the subscale ‘social and family connectedness’ and PCS (0.06, *p* > 0.05).

**Table 3:** Pearson correlations of 8-factor Holistic Health Status Questionnaire with SF-12 Health Survey

	HHS components								
	Psychological expression	Physical symptoms	Changes in self and family	Fatalism	Social and family connectedness	Self-query	Religion and faith	Coping style	HHS
HHS components									
Psychological expression									
Physical symptoms	.686^**^								
Changes in self and family	.681^**^	.716^**^							
Fatalism	.208^**^	.270^**^	.258^**^						
Social and family connectedness	.243^**^	.157^**^	.058	−.074					
Self-query	.330^**^	.229^**^	.253^**^	.157^**^	.029				
Religion and faith	.173^**^	.078	.206^**^	−.077	.233^**^	−.034			
Chinese coping skills	.253^**^	.125^*^	−.016	−.126^*^	.257^**^	.125^*^	.090		
HHS	.899^**^	.859^**^	.836^**^	.366^**^	.287^**^	.395^**^	.267^**^	.245^**^	
SF12									
PCS	.258^**^	.533^**^	.583^**^	.173^**^	.057	.118^*^	.126^*^	−.116^*^	.464^**^
MCS	.671^**^	.558^**^	.559^**^	.239^**^	.247^**^	.237^**^	.177^**^	.231^**^	.698^**^

*HHS* Holistic Health Status Questionnaire, *SF12* SF-12 Health Survey

*p < 0.05

**p < 0.01

## Discussion

The purpose of this study was to develop and evaluate the psychometric properties of the HHSQ. Findings yielded an initial factor structure of the HHSQ through EFA with acceptable internal consistency and satisfactory convergent validity. The lack of a tool for assessing holistic health using individuals’ direct expressions highlights an important gap in the research in this area. This initial development of the HHSQ is the first step in the task of filling this gap, which has cultural and linguistic relevance. It is a promising tool for use after future scale refinement and validation.

The HHSQ consists of the domains of psychological expression, changes in self and family, physical symptoms, social and family connectedness, fatalism, religion and faith, self-query, and coping style. It contains not only the bio-psycho-social and spiritual aspects as denoted in the generic frame of holism, but is also characterized by elements in relation to chronic illness, featuring Chinese cultural attributes and expressions. Though the sample subjects in this study are of Chinese origin, the attributes and expressions are congruent with the literature to be discussed below, which includes populations other than the Chinese.

Spirituality leads to finding a purpose and meaning in life that is attributed to human existence [55]. Being disabled after an illness is a traumatic event, and the phenomenon of searching for meaning after a critical event is commonly reported in both Eastern and Western literature [24, 56–58]. Individuals who were chronically ill often raised questions about their own identities in their life stance, trying to find answers and seek some purpose in what happened to change their health. Individuals sometimes interpreted the meaning of their experience in relation to nature, fate or a higher power [57, 59]. This is consistent with our study findings, in which individuals became fatalistic, relating/turning to religion and seeking a sense of hope when faced with the harsh reality of being disabled [60–62]. Items such as ‘I believe it is fate’, ‘I believe that it is predestined’, ‘I find it helpful to pray to God/gods/my religion’ and ‘There is hope in my future’ were the expressions that demonstrated this phenomenon.

The integrated beliefs of Confucianism, Buddhism and Taoism inspire the concept of holism in Chinese societies [26–29]. An individual self is constructed in webs of relationships, as found in the Confucian ‘five basic relationships’ tradition [26, 63]. Three out of these five basic relationships occur within the family: father-son, husband-wife, and elder-younger. The individual is traditionally held with close relatedness within the family, where the holism (wholeness) of an individual in Chinese culture is contextualized. Inner and deep feelings can only be shared with those with whom a person has close ties [64]; in particular, the emotional bond with family members is salient [30]. The importance of family connectedness and the important cultural roles assumed by Chinese families in taking care of sick members, as reported in this sample of HK Chinese, demonstrate the relatedness of the individual with the family during the course of illness, which underpins the concept of wholeness of an individual. Items reported from our sample, such as ‘My family gives me great comfort’ and ‘My family cares a lot about me’, reflect the importance of this aspect.

The styles of coping and living with chronic diseases among the HK Chinese participants were shaped by traditional Chinese values and beliefs. The attitude of ‘letting go’ was one of the coping styles. Although ‘letting go’ is a common response found in both eastern and western literature [16, 27, 65], the meaning of ‘letting go’ among Chinese is derived from Confucian, Buddhist and Taoist beliefs, namely that illness is part of life, and over-attachment to an illness event will lead to suffering. Freedom from emotional turmoil and maintaining peace, balance and harmony to keep goodness in mind-body-spirit in order to be more resilient in the course of suffering could transcend to a coping attitude of non-action, which is valued in Taoism [26, 58, 66]. As such, Chinese participants might exhibit a ‘trying not to think too much’ attitude (a way of being unconcerned) and try to learn to live with their reality. The coping style of accepting reality could possibly be interpreted as a passive acceptance of reality when circumstances are beyond their personal control or when they are under physical constraints such as living with permanent physical disabilities. The individuals had not actually taken active measures to solve the problem or make changes. This might account for our study finding that those with higher scores of coping style in the HHSQ had lower SF-12 Health Survey PCS (physical component summary) scores. The study by Siu et al. [67] also revealed that the Confucian virtue of forbearance may have negative consequences for health and well-being, with passive adaptive coping (i.e. accepting reality, letting fate have its way) correlated with more reported physical and behavioral symptoms.

It has been discussed that the same data, after extraction with several different techniques and followed by varimax rotation, resulted in similarities among factor solutions. The choice between using principal component analysis (PCA) and principal factor analysis (PFA) depends on the assessment of the fit between models, the data set, and the goals of the research [52]. The approach employed in this study was sensible, based on the premise that an EFA using PCA and varimax rotation yielded factors that were congruent with the conceptual understanding of holistic health. The final factor model explaining 56.38 % of the variance was considered acceptable [68]. If an item did not effectively measure the factor of interest, it should be removed to improve the factor content validity [52, 69]. Seven items were removed from the factor analysis as they were less congruent with the factor content or marker items of their respective factors. Almost all items in the final model were significantly loaded to a factor (factor loading of 0.3 or above) [69, 70], indicating that these items were effective indicators representing the interest of their respective factors.

The internal consistency indicated acceptable values according to the set criterion for a newly-developed instrument. Although item-to-subscale correlations above 0.3 are usually considered good [71], others have suggested that the item-to-subscale correlation can be above 0.2 [72] or even 0.15 [73]. The findings of item-to-subscale correlations in the present study were considered acceptable. Of the 45 items, only one, ‘There is hope in my future!’, under factor 6 ‘religion and faith’, obtained a low correlation (0.17) and might require further exploration in future scale validation or refinement. Though the subscale-to-total correlations, factor 6 ‘religion and faith’, and factor 8 ‘coping style’ had relatively low correlations, the findings revealed that each of them contained three items with significant factor loadings that are generally needed [68, 70, 74]. It is plausible to expect items to be less homogenous within a three-item domain because the stability of a domain can be caused by an increase in the number of significant items, reflecting the depth and breadth dimensions of the domain. However, Factors 6, 7 and 8 had an acceptable mean inter-item correlation. When there are a small number of items in the domain, it may be better to report the mean inter-item correlation for the items regarding the domain homogeneity, and an optimal mean inter-item correlation values range from 0.2 to 0.4 is recommended [75]. Factors 6, 7 and 8 had a mean inter-item correlation of 0.38, 0.29 and 0.33, respectively. Based on the Chinese cultural and conceptual perspectives, these factors were relevant for retention in the model. As an increase in the number of significant items reflecting the depth and breadth dimensions of a domain improves factor clarity or stability as well as enhancing subscale-to-total correlations, the domains of religious beliefs and coping style among Hong Kong Chinese people need to be further explored for future scale refinement.

Although factor analysis plays a unique role in scale development, the subjective and judgmental nature of decisions made during the analysis process is often the basis for serious criticism, thus prior knowledge about the research area is crucial in the process [44]. The present study, based on understanding from the literature and expert input from health professionals, and informed by the findings of the qualitative study, generated items that were subjected to testing for factor analysis. Conservatively setting the factor loading at a minimal 0.30 as a significant criterion level prevented the inappropriate dropping of items or factors in the early stage of scale development using exploratory factor analysis. The conceptual guide and qualitative findings helped further support the decisions during the factor analysis procedures. Given that the first factor, accounting for 25 % of the variance, represented chiefly the psychological dimension, we decided to include factors of lesser variance as they contained the bio-psycho-social and spiritual aspects as denoted in the generic frame of holism, and these factors were consistent with the Chinese cultural and conceptual perspectives. Undoubtedly, further studies with larger sample sizes are needed to improve the depth and breadth dimensions for the factors with smaller variances.

The optimal holistic health and well-being of an individual are aligned with their experiencing a better quality of life. Satisfactory construct validity of the HHSQ was found in the convergent validity, with a significant correlation between the overall HHSQ and the two main domains, PCS and MCS, of the quality of life measure, the SF-12 Health Survey. It should be noted that weak relationships were found between some individual factors of the HHSQ and the quality of life measure. The measure of holistic health, however, unlike SF-12, does not measure the compartmentalized dimensions of the physical and mental domain. Rather, the HHSQ aims at reporting the respondents’ state of health using descriptions that are grounded in their lived experiences and world view [35–37].

It is interesting to note that the statements generated from the respondents in this study corresponded with those reported in the study by Faull [36] conducted in New Zealand. Both groups expressed holistic health concerns in terms of one’s relationship with oneself, one’s family, the Higher Being and healthy functioning [36, 76]. In this study, however, the Chinese respondents tended to suggest perspectives that were related to fate, letting go and passive acceptance. These measures have been described as a uniquely Chinese way of coping in yielding to predestiny (feng-shui) and serendipity (yuan-fen) [67]. The HHSQ has proven to be an instrument with established psychometric properties that help in understanding Chinese perceptions of holistic health, particularly for those with chronic illnesses.

### Limitations and implications for the research

The present study has limitations. While taking account of patients’ health conditions and the feasibility of recruiting participants in a sufficiently large sample, this study was limited to recruiting a proportionate sample of participants with different kinds of chronic diseases, although efforts were made to perform wide-scale recruitment of participants who were all chronically ill and required holistic care. It was a drawback that the subjects used in this study to develop and evaluate the scale were very different in many ways in terms of religious background, type and stage of chronic disease, and health condition; therefore the sample was not fully representative of the population we were trying to target with the instrument. The purposive and convenience samples added further limitations to the generalizability of the findings, as they might have inadvertently excluded some groups of respondents. Given that the qualitative sample recruited post-stroke participants, its criteria might have been too narrow to enable the sample to be generalized to other chronic illnesses, and thus certainly might not reflect the ultimate interest in those with other chronic diseases. However, having considering that specific measures are more sensitive for the disease of interest, while generic measures help to compare health-related quality of life (HRQOL) among different diseases, our measure generated in stage 1 interestingly conveyed no specific description of the illness concerned, but contained general expressions of holistic concern after an episode of chronic illness. These findings from using heterogeneous samples with various kinds of chronic diseases have successfully derived a factor model accounting for over 56 % of total explained variance with the established reliability and validity of the HHSQ instrument for expressing generic measures of holistic health. Future research needs to be conducted to test the sensitivity of this generic measure and establish its content validity in assessing the well-being of clients with specific diseases of interest.

Using the SF-12 Health Survey in the convergent validity, it fell way short of validating the spiritual components of the HHSQ since SF-12 does not have an explicit spiritual dimension in its measure. Validating the HHSQ in the future might mean administering it along with the scales that will address the specific spiritual dimension. While our findings entailed the discussion of “fate” and “praying to God/gods”, which echoed two of the external health loci of control dimensions – the belief in chance and the God locus of health control, scales focused on a multidimensional health locus of control might be appropriate for future validation of the HHSQ.

The authors also attempted to evaluate the factor structure of the HHSQ using confirmatory factor analysis in one of the samples in the second stage of the study, but the 8-factor, 45-item HHSQ did not provide a satisfactory fit to the data – the goodness-of-fit indices of <0.90 and the value of the root mean square error of approximation (RMSEA) were not within the range of 0.05–0.08. The study also fell short of recruiting a sufficient number of participants to take part in a two-week test-retest stability check. Although test-retest stability had shown an intra-class coefficient (ICC) of 0.85, it was based on far too few participants (*n* = 7) to give meaningful stability data. Response biases such as those inherent in the self-reporting survey might be a limitation, hence the possibility of over- and under-reporting biases toward desirable and less acceptable responses respectively should be considered. This study only provides initial psychometric findings on the HHSQ, but future studies are warranted to further validate and improve the scale.

The researchers employed the Classical Test Theory (CTT) approach in analyzing the data. The Item Response Theory (IRT) has advantages over the CTT in estimating health outcomes since it has more vigorous assumptions in data fit and is less sample-dependent [77]. The IRT is also more sensitive in detecting change in health over time [78]. However, the IRT requires a larger sample size (usually over 500) than the CTT (200 to 500 in general) for item parameter estimation [77]. Our study has a sample size of just over 300, so it is difficult to perform an accurate item calibration using the IRT for the existing sample.

## Conclusion

The increased prevalence of chronic diseases is a global health issue. Once an individual has been diagnosed with a chronic disease, healthcare support in different aspects is essential. In this regard, a culturally-sensitive and context-relevant assessment tool and an evaluation of holistic health are central to healthcare practice. This study has taken a step towards exploring the conceptual holistic framework relevant to healthcare in the management of chronic disease, and operationalizing it. The 8-factor, 45-item HHSQ was initially developed in this study - this holistic care measurement might stimulate more studies although the instrument itself might not be mature enough. It appears promising for use in research to assess holistic health status, as it taps into the relational experiences and connectedness within the bio-psycho-social-spiritual dimensions of a person with chronic disease. The HHSQ has the potential to be used in all Chinese-speaking populations with its established culture relevance. While this instrument may contain expressions of well-being that are more emphasized by the Chinese, such as the concept of karma, there are also other more generic items that are shared by non-Chinese respondents, such as feeling confused. It will also be interesting to translate this instrument with confirmed language equivalence to make cross-cultural comparisons of holistic health. The language used in this instrument is derived from the subjective expressions of patients who have experienced living with chronic illness.
